# High-Level Genetic Diversity and Complex Population Structure of Siberian Apricot (*Prunus sibirica* L.) in China as Revealed by Nuclear SSR Markers

**DOI:** 10.1371/journal.pone.0087381

**Published:** 2014-02-07

**Authors:** Zhe Wang, Ming Kang, Huabo Liu, Jiao Gao, Zhengdong Zhang, Yingyue Li, Rongling Wu, Xiaoming Pang

**Affiliations:** 1 National Engineering Laboratory for Tree Breeding, Key Laboratory of Genetics and Breeding in Forest Trees and Ornamental Plants, Ministry of Education, Center for Computational Biology, College of Biological Sciences and Biotechnology, Beijing Forestry University, Beijing, China; 2 South China Botanical Garden, Chinese Academy of Sciences, Guangzhou, China; USDA-ARS-SRRC, United States of America

## Abstract

Siberian apricot (*Prunus sibirica* L.), an ecologically and economically important tree species with a high degree of tolerance to a variety of extreme environmental conditions, is widely distributed across the mountains of northeastern and northern China, eastern and southeastern regions of Mongolia, Eastern Siberia, and the Maritime Territory of Russia. However, few studies have examined the genetic diversity and population structure of this species. Using 31 nuclear microsatellites, we investigated the level of genetic diversity and population structure of Siberian apricot sampled from 22 populations across China. The number of alleles per locus ranged from 5 to 33, with an average of 19.323 alleles. The observed heterozygosity and expected heterozygosity ranged from 0.037 to 0.874 and 0.040 to 0.924 with average values of 0.639 and 0.774, respectively. A STRUCTURE-based analysis clustered all of the populations into four genetic clusters. Significant genetic differentiation was observed between all population pairs. A hierarchical analysis of molecular variance attributed about 94% of the variation to within populations. No significant difference was detected between the wild and semi-wild groups, indicating that recent cultivation practices have had little impact on the genetic diversity of Siberian apricot. The Mantel test showed that the genetic distance among the populations was not significantly correlated with geographic distance (r = 0.4651, p = 0.9940). Our study represents the most comprehensive investigation of the genetic diversity and population structure of Siberian apricot in China to date, and it provides valuable information for the collection of genetic resources for the breeding of Siberian apricot and related species.

## Introduction

Siberian apricot (*Prunus sibirica* L.), an ecologically and economically important tree species, is widely distributed across the mountainous areas of northern and northeastern China, eastern Siberian, and Mongolia [1]. It can adapt to a variety of harsh environmental conditions, including cold stress, drought stress, and reduced soil fertility, making it one of the primary choices for controlling desertification in northern and northwestern China. Siberian apricot almond is not only a traditional dry food, but also an important raw material for food, cosmetics, and biodiesel manufacturing. Thus, Siberian apricot is important to the income of farmers in these areas [2], [3].

In recent decades, almond products have become increasingly popular on the domestic and international market. Consequently, many almond processing plants have been established around the major areas of production in China. However, Siberian apricot resources are declining due to backward management patterns and deterioration of the natural environment [4]. Furthermore, diseases and insect pests such as awning caterpillar (*Malacosoma neustria testacea Motsch*) and leaf roller (*Adoxophyes honmai*) have made the originally fragile natural environment even worse [5]. Despite the hardiness of Siberian apricot, its flowers will wither if a late frost hits during flowering, and this can cause a serious reduction in yield or no yield at all. Therefore, there is an urgent need to develop a Siberian apricot cultivar with increased tolerance to both abiotic and biotic stresses. The success of breeding programs is based on the knowledge and availability of genetic variability for efficient selection [6]. However, Siberian apricot, as a building block for breeding programs, has not been extensively studied in China until now.

Increased knowledge of the genetic diversity and population structure of Siberian apricot in China will provide the basis for protecting, utilizing, and improving our resources. Therefore, an assessment of the extent and nature of the genetic variation in Siberian apricot is important for breeding and genetic resource conservation programs. Traditionally, genetic diversity has been assessed based on morphological characteristics, which are often influenced by the environmental conditions. With the advent of molecular markers, including restriction fragment length polymorphisms, amplified fragment length polymorphisms, simple sequence repeats (SSRs), and single nucleotide polymorphisms, much progress has been made in understanding the genetic diversity and population structure of various species [7]–[10]. Among these markers, SSRs have been the first choice for the study of genetic diversity and population structure owing to their desirable genetic attributes, including high numbers of polymorphisms, wide genomic distribution, co-dominant inheritance, and high degree of reproducibility [11], [12]. Nuclear SSR makers have also proven to be very useful for the evaluation of genetic diversity in apricot [13], [14]: they have been employed to investigate the genetic diversity of Siberian apricot in the Yan Mountains of China [15]. However, a comprehensive analysis of Siberian apricot genetic diversity and its population structure in China at the DNA level is lacking.

In this study, 31 nuclear SSR loci developed previously for this species [16] were used to analyze the genetic diversity and structure of Siberian apricot populations in China. The objectives of the study were to provide a complete picture of the organization of genetic diversity of Siberian apricot populations in China and to reveal the origin of the genetic variation in Siberian apricot populations.

## Materials and Methods

### Sampling

**Figure 1:**
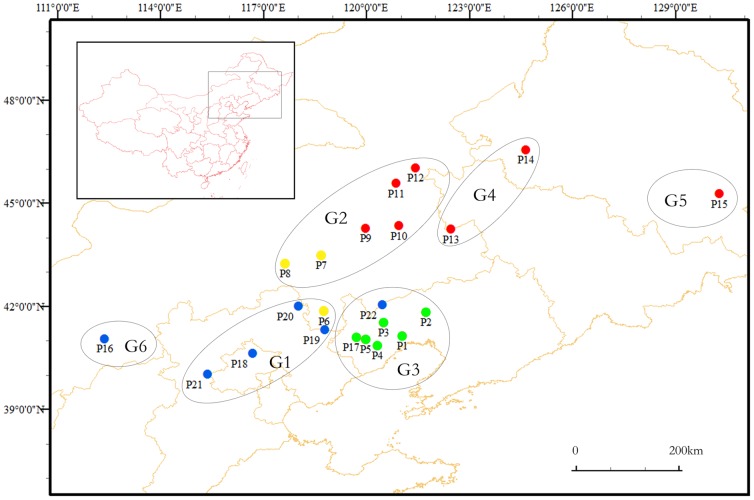
Geographic distribution of the analysed Siberian apricot sampling in China. The image was generated by the software ArcGIS (ESRI, Redlands, CA, USA). Green dots represent genetic cluster C1, yellow dots represent genetic cluster C2, red dots represent genetic cluster C3, blue dots represent genetic cluster C4.

A total of 672 individuals of Siberian apricot representing 22 populations were collected throughout the areas of distribution in China (Table 1). A total of 25 to 32 individuals were sampled for each population, and the coordinate of each tree was recorded using a global positioning system. The distance between any two individuals at each location was >50 m. The 22 populations were from 21 sampling locations (P5 and P17 were from the same region) across 18 longitudes in the east-west direction and across 6 latitudes in the north-south direction. The highest altitude of the locations was 1,334 m (P20), while the lowest altitude was 87 m (P1). Daqing (P14) had a minimum altitude gap of only 3 m, while Weichang (P20) had a maximum altitude gap of 271 m. The sampled populations were divided into six groups according to their geographical locations. The Yan Mountains group (G1) included P7, P8, P9, P10, P11, and P12; the Greater Khingan Mountains group (G2) included P18, P19, P20, P21, and P6; the Western Liaoning Hills group included (G3) P1, P2, P3, P4, P5, P17, and P22; the Northeast Plain group (G4) included P13 and P14; the Linkou group (G5) included P15; and the Daqingshan Mountain group (G6) included P16 (Figure 1). No specific permits were required for this field study. All sampling locations were public space where anyone can enter and collect forest products, regardless of ownership. In addition, the field study did not involve endangered or protected species.

**Table 1:** Summary of Siberian apricot sampling locations around China.

Population	Population ID	Sample size	Locality	Elevation (m)	Origin
Jinzhou, Liaoning	P1	31	N41°09′ E121°03′	87∼226	Wild
Fuxin, Liaoning	P2	30	N41°50′ E121°44′	384∼456	Semi-wild
Chaoyang, Liaoning	P3	30	N41°32′ E120°30′	361∼533	Wild
Huludao, Liaoning	P4	31	N40°52′ E120°19′	237∼267	Wild
Kazuo, Liaoning	P5	30	N41°03′ E119°58′	530∼736	Wild
Jinshan, Inner Mongolia	P6	31	N41°52′ E118°46′	998∼1126	Wild
Daban, Inner Mongolia	P7	32	N43°30′ E118°41′	681∼780	Semi-wild
Jingpeng, Inner Mongolia	P8	29	N43°15′ E117°38′	1092∼1275	Wild
Tianshan, Inner Mongolia	P9	32	N44°17′ E119°58′	486∼519	Wild
Lubei, Inner Mongolia	P10	32	N44°21′ E120°56′	301∼319	Wild
Tuliemaodu, Inner Mongolia	P11	32	N45°35′ E120°52′	477∼564	Wild
Keqinzhongqi, Inner Mongolia	P12	32	N46°02′ E121°26′	475∼584	Wild
Baicheng, Jilin	P13	32	N44°15′ E122°27′	158∼167	Wild
Daqing, Heilongjiang	P14	32	N46°34′ E124°39′	147∼150	Wild
Linkou, Heilongjiang	P15	30	N45°17′ E130°17′	255∼316	Wild
Wulancabu, Inner Mongolia	P16	31	N41°04′ E112°22′	157∼195	Wild
Kazuo, Liaoning	P17	30	N41°06′ E119°43′	317∼364	Semi-wild
Huairou, Beijing	P18	30	N40°38′ E116°41′	426∼507	Wild
Pingquan, Hebei	P19	29	N41°19′ E118°47′	641∼733	Wild
Weichang, Hebei	P20	31	N42°01′ E118°01′	1063∼1334	Wild
Zhuolu, Hebei	P21	30	N40°02′ E115°22′	1149∼1222	Wild
Chifeng, Inner Mongolia	P22	25	N42°03′ E120°27′	693∼821	Wild

In China, Siberian apricot has been cultivated for decades in an experimental forest. Currently, the main method of propagation is to sow seeds collected from the immediate area or near the region without selection. Three such populations were collected and designated as semi-wild type. All other populations were from the wild (Table 1). Young leaves were collected and placed immediately in Ziploc bags preloaded with colored silica gel to dry them and preserve them for DNA extraction.

### Microsatellite DNA Analysis

Total genomic DNA was extracted from dry leaves collected from all localities using a modified version of the cetyl trimethylammonium bromide method [17]. The quality and concentration of the extracted DNA was determined by 1% agarose gel electrophoresis and ultraviolet spectrophotometry.

Thirty-one microsatellite loci were employed to study the genetic diversity on wild Siberian apricot accessions including 23 recently developed in Siberian apricot [16], [18], one from apricot (*Prunus armeniaca* L.) [19] and seven from peach (*Prunus persica* L.) [20]–[22] (Table S1). The forward primer of each pair was tagged with a section of the universal M13 sequence (5′-TGTAAAACGACGGCCAGT-3′) during synthesis. Amplification was performed in a 10-µL reaction mixture containing 1 µL of DNA template (10 ng/µL), 5 µL of 2X Taq mix, 0.4 µL of the forward primer (1 µM), 1.6 µL of the reverse primer, 1.6 µL of M13 primer (1 µM) with a fluorescent label (FAM, HEX, ROX, or TAMRA), and 1.4 µL of ddH_2_O. The reaction conditions were: 94°C for 5 min, followed by 30 cycles of 94°C for 30 s, 55°C for 30 s, and 72°C for 30 s, followed by 8 cycles of 94°C for 30 s, 53°C for 40 s, and 72°C for 30 s, with a final extension at 72°C for 10 min. The products were separated in an ABI 3730×L DNA Analyzer using GeneScan-500LIZ as an internal marker (Applied Biosystems, Foster City, CA, USA). The amplicon fragments were sized using Gene-Marker 1.75 software (SoftGenetics LLC, State College, PA, USA). All rare alleles and private alleles were re-amplified. For the alleles from the homozygous loci, the purified PCR products were sent to sequence. For the alleles from the heterozygous loci, the targeted fragments were separated, cloned and sequenced following the protocol by Chen et al [23]. These sequences were compared with target fragments to distinguish whether they were non-specific amplifications.

### Data Analysis

FLEXBIN was used for automated binning of the microsatellite raw data [24], and the Excel Microsatellite Toolkit [25] was employed to convert the size data into various formats for further analysis. The level of genetic diversity was estimated using GENALEX software version 6.41 [26] with the following statistics: number of alleles (Na), effective number of alleles (Ne), Shannon’s Information Index (I), observed heterozygosity (Ho), expected heterozygosity (He) [27], and F-statistics calculations (F_IS_, F_IT_, and F_ST_).

Clustering based on a Bayesian model was used to evaluate the genetic structures of the Siberian apricot populations with the software package STRUCTURE [28] in its extended version 2.3.3 [29], [30]. The admixture model and independent allelic frequencies were employed to analyze the data set without prior population information. The length of the burn-in period and number of MCMC reps after burn-in were set to 25,000 and 100,000, respectively. These steps were used to determine the ancestry value, which estimates the proportion of an individual’s genome that originated from a given genetic group. The algorithm was run ten times for each K value, from 1 to 22. Using an ad hoc quantity constructed from the second-order rate of change of the likelihood function with respect to K (ΔK), the distribution of ΔK showed a clear peak at the true value of K [31].

The observed genetic variation among and within the populations and genetic groups was characterized by an analysis of molecular variance (AMOVA) using ARLEQUIN version 3.5 [32]. This analysis subdivided the 22 populations into two different origin groups, six geographical groups and K groups. Three hierarchical divisions were identified based on the genetic variance: within populations, among populations within groups, and among groups using a nonparametric permutation procedure incorporating 10,000 iterations. In addition, we tested all of the loci for deviations from Hardy-Weinberg equilibrium (HWE) using ARLEQUIN version 3.5 [33] with 100,000,000 steps in the Markov chain [34] and 100,000 dememorization steps. We selected F_ST_ and R_ST_ to calculate the genetic differentiation of all population pairs. The values of F_ST_ and R_ST_ were calculated using FSTAT version 2.9.3 [35] and ARLEQUIN version 3.5 [33], respectively. To examine the effect of geographic distance on genetic structure, correlations between the pairwise genetic distances, represented by F_ST_/(1−F_ST_) estimates [36], and pairwise geographic distances among 19 wild populations, which were calculated according to the latitude and longitude of each site with Vincenty’s formula (http://www.movable-type.co.uk/scripts/latlong- vincenty.html), were tested using the Mantel test implemented by Isolation By Distance Web Service version 3.23 (http://ibdws.sdsu.edu/~ibdws/) [37], [38]. We also employed Monmonier’s maximum difference algorithm to highlight geographical features corresponding to pronounced genetic discontinuity using BARRIER version 2.2 [39].

## Results

### Genetic Diversity Among the Loci

A total of 31 microsatellite loci were used to genotype 672 individuals of Siberian apricot (Table 2). The genetic profiles detected 599 alleles, which ranged between 5 and 33 per locus, 207 of which were rare alleles with a frequency below 1%. The sequencing results showed that all the rare alleles were true alleles with the sequences containing expected microsatellites. The Ne in the total samples was 6.804 (range, 1.041 to 13.653). I ranged from 0.119 to 2.987, with an average of 2.062. The Ho in the total sample was 0.639, which deviated from the He (0.774). Genetic subdivision using F-statistics estimated a moderate inbreeding coefficient (0.173) and an F_ST_ value of 0.071 across all loci, indicating moderate genetic differentiation among the sites. The values of gene flow ranged from 1.869 to 8.224, with an average of 3.595. Most loci conformed to HWE and no population had a particularly large number of loci that deviated from HWE.

**Table 2:** Diversity indices of 31 nuclear microsatellite loci from data of 672 individuals.

Locus	Na	Ne	I	Ho	He	Fis	Fit	Fst	Nm	Rare alleles
PSL1	16	4.652	1.837	0.600	0.785	0.181	0.238	0.070	3.334	6
PSL3	7	1.553	0.781	0.308	0.356	0.073	0.137	0.069	3.361	3
PSL6	26	9.485	2.628	0.689	0.895	0.179	0.228	0.059	3.952	7
PSL7	23	10.513	2.577	0.807	0.905	0.047	0.113	0.069	3.376	8
PSL8	11	3.378	1.342	0.617	0.704	0.036	0.121	0.089	2.567	7
PSL10	19	4.744	1.928	0.733	0.789	-0.049	0.074	0.118	1.869	9
PSL11	24	11.934	2.681	0.790	0.916	0.077	0.154	0.083	2.758	7
PSL12	21	9.535	2.529	0.816	0.895	0.034	0.092	0.060	3.929	4
PSL13	20	3.528	1.816	0.654	0.717	0.028	0.086	0.059	3.970	11
PSL14	12	2.261	1.295	0.518	0.558	0.034	0.074	0.042	5.765	4
PSL16	24	2.924	1.899	0.313	0.658	0.485	0.523	0.074	3.108	7
PSL18	27	12.249	2.765	0.781	0.918	0.094	0.150	0.062	3.808	10
A1-10	20	6.345	2.293	0.388	0.842	0.513	0.548	0.071	3.253	6
A3-9	8	3.907	1.470	0.388	0.744	0.421	0.484	0.109	2.045	3
A3-66	27	11.305	2.770	0.770	0.912	0.103	0.157	0.060	3.931	6
H1-7	23	10.389	2.606	0.838	0.904	0.000	0.072	0.072	3.205	6
H1-11	17	5.955	2.154	0.711	0.832	0.076	0.148	0.079	2.931	5
H1-77	33	13.653	2.987	0.874	0.927	0.016	0.056	0.041	5.891	7
H1-87	23	10.363	2.589	0.798	0.904	0.047	0.118	0.075	3.080	7
H2-11	14	4.725	1.806	0.814	0.788	-0.078	-0.033	0.042	5.641	5
H2-27	12	3.222	1.536	0.653	0.690	-0.030	0.052	0.080	2.887	4
H2-77	31	13.117	2.864	0.475	0.924	0.455	0.497	0.077	2.981	11
H2-79	19	5.487	2.119	0.779	0.818	-0.010	0.050	0.060	3.940	7
BPPCT 002	29	7.198	2.352	0.799	0.861	0.004	0.073	0.069	3.374	16
BPPCT 030	12	5.825	1.945	0.731	0.828	0.024	0.121	0.100	2.253	3
CPPCT 6	17	9.249	2.386	0.831	0.892	0.005	0.068	0.063	3.714	4
UDP96-001	5	1.041	0.119	0.037	0.040	0.030	0.059	0.029	8.244	3
UDP96-005	22	4.872	2.140	0.388	0.795	0.474	0.515	0.079	2.923	10
UDP96-010	12	1.844	0.975	0.437	0.458	-0.019	0.043	0.061	3.859	7
UDP98-412	19	9.794	2.523	0.856	0.898	-0.026	0.049	0.073	3.180	2
ssrPaCITA 15	26	5.871	2.210	0.621	0.830	0.212	0.288	0.097	2.317	12
**Mean**	19.323	6.804	2.062	0.639	0.774	0.111	0.173	0.071	3.595	6.677
**Total**	599									207

Na: Number of Different Alleles; Ne: Number of Effective Alleles; I: Shannon’s Information Index; Ho: Observed Heterozygosity; He: Expected Heterozygosity; F_IS_: inbreeding coefficient; F_IT_: over inbreeding coefficient; F_ST_: fixation index; Nm: Gene Flow.

### Genetic Structure of the Siberian Apricot Samples

**Figure 2:**
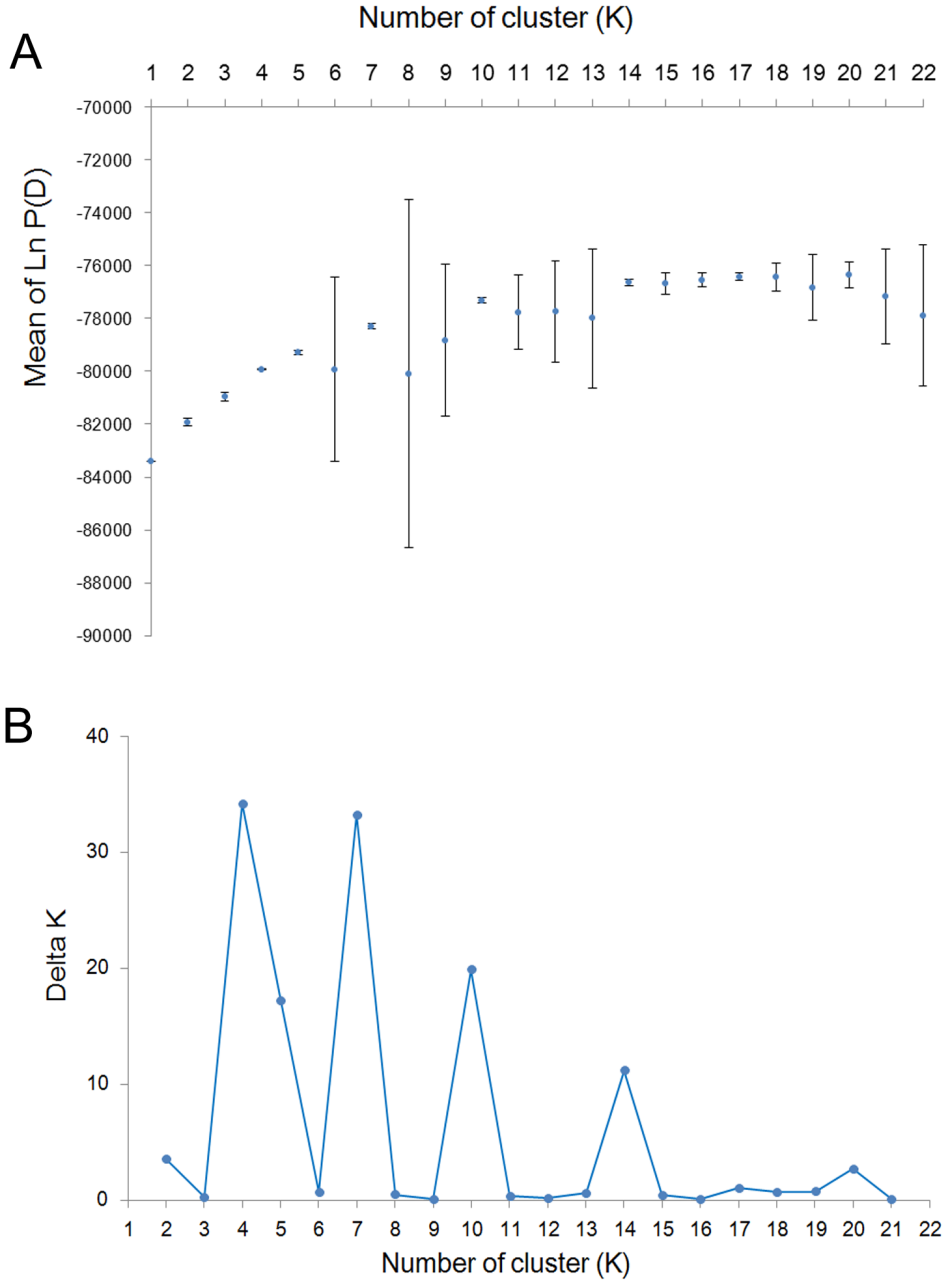
Plot of the Ln P(D) ± SD and delta K (ΔK). The mean of Ln P(D) was based on ten repetitions for each K value.

**Figure 3:**
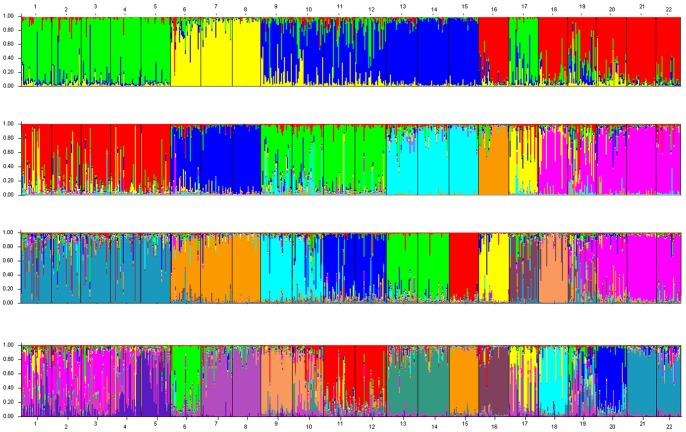
Clustering of 22 Siberian apricot populations. Each individual is shown as a vertical line divided into segments representing the estimated membership proportion in the four, seven, ten and fourteen ancestral genetic clusters inferred with STRUCTURE.

The genetic structure of the Siberian apricot samples was investigated by a Bayesian-based population assignment analysis using STRUCTURE [28]. Our results show a clear maximum for ΔK at K = 4 (Figure 2B), in which all individuals were classified into four different clusters. About 80% individuals belonged to each genetic cluster, which showed strong ancestry values with an average >0.90 (Table S2). Regarding the genetic cluster 1 (C1) which included P1, P2, P3, P4, P5 and P17, only 14 individuals (7.4%) showed ancestry values <0.60. Eighteen individuals which were from the locations belonged to other genetic clusters. These individuals corresponded to two accessions from P9, two accessions from P10, two accessions from P11, six accessions from P12, one accession from P18, and five accessions from P19 (Figure 3 and Table S2). The genetic cluster 2 (C2) consisted of P6, P7 and P8, and only 13 individuals (13.1%) showed ancestry values <0.60. We found 10 individuals were from the locations belonged to other genetic clusters for C2. These individuals corresponded to two accessions from P2, one accession from P9, three accessions from P10, one accession from P11, one accession from P19, and two accessions from P20. Within the genetic cluster 3 (C3), which contained population P9, P10, P11, P12, P13, P14 and P15, only 25 individuals (11.6%) showed ancestry values <0.60, and 11 individuals were from the locations belonged to other genetic clusters. These individuals correlated to one accession from each of the population P1, P2, P3 and P6, five accessions from P17, and two accessions from P19 (Figure 3 and Table S2); All remaining populations including P16, P18, P19, P20, P21 and P22 were clustered into the genetic cluster 4 (C4), among which 16 individuals (9.5%) showed ancestry values <0.60. And only two individuals were from P6 which belonged to C2 (Figure 3 and Table S2).

At the same time, the second largest ΔK at K = 7 was much larger than the remaining values. In addition, two clear peaks were observed at K = 10 and 14 (Figure 2B). When K = 7, P16 and P17 were separated into two new genetic clusters from genetic cluster C1 and genetic cluster C2, while genetic cluster C3 was divided into two genetic clusters. On the basis of seven genetic clusters, the 3th, 4th and 7th genetic clusters, were all split into two genetic clusters while K = 10. When k = 14, the 1st and the 2nd genetic clusters were further divided into two genetic clusters, and the 10th genetic cluster was divided into three detailed genetic clusters on the basis of the clustering of ten genetic clusters (Figure 3).

### Genetic Diversity Among the Siberian Apricot Populations

The population genetic parameters used in this study are summarized in Table 3. The highest degree of genetic diversity occurred in P4 (Ne = 6.084, Ho = 0.672, and He = 0.753), P10 (Ne = 5.709, Ho = 0.705, and He = 0.763), and P17 (Ne = 5.700, Ho = 0.666, and He = 0.769), while the diversity was lowest in P8 (Ne = 4.155, Ho = 0.592, and He = 0.674), P16 (Ne = 3.544, Ho = 0.564, and He = 0.632), and P21 (Ne = 4.163, Ho = 0.558, and He = 0.658). P16 and P21 were unique populations with3 and 4 more private alleles respectively, although they had the lowest genetic diversity.

**Table 3:** Genetic diversity estimations in wild and semi-wild groups, genetic clusters and all populations.

Pop	Sample size	Na	Ne	Ho	He	F	Private alleles
C1	182	16.645	6.583	0.653	0.768	0.147	32
C2	92	11.903	5.221	0.642	0.721	0.106	5
C3	222	15.806	6.630	0.668	0.780	0.141	29
C4	176	15.258	5.609	0.583	0.733	0.194	26
Wild	580	18.968	6.767	0.638	0.773	0.171	152
Semi-wild	92	16.694	6.607	0.643	0.770	0.161	11
Pop1	**31**	10.742	5.351	0.661	0.733	0.087	7
Pop2	**30**	10.355	5.465	0.641	0.736	0.122	3
Pop3	**30**	10.129	5.370	0.618	0.710	0.122	1
Pop4	**31**	11.226	6.084	0.672	0.753	0.123	6
Pop5	**30**	9.806	5.708	0.650	0.744	0.137	3
Pop6	**31**	8.742	4.609	0.671	0.704	0.047	2
Pop7	**32**	8.774	4.524	0.631	0.702	0.101	2
Pop8	**29**	7.419	4.155	0.621	0.674	0.076	1
Pop9	**32**	10.226	5.099	0.652	0.734	0.115	3
Pop10	**32**	10.484	5.709	0.705	0.763	0.071	3
Pop11	**32**	10.032	5.572	0.666	0.750	0.116	1
Pop12	**32**	9.935	5.165	0.657	0.738	0.104	0
Pop13	**32**	9.871	5.436	0.652	0.736	0.117	2
Pop14	**32**	9.806	5.114	0.661	0.725	0.086	0
Pop15	**30**	8.290	4.921	0.683	0.740	0.082	4
Pop16	**31**	7.613	3.544	0.564	0.632	0.099	3
Pop17	**30**	11.000	5.700	0.666	0.769	0.132	6
Pop18	**30**	8.226	4.648	0.592	0.678	0.122	1
Pop19	**29**	10.000	5.264	0.641	0.734	0.121	3
Pop20	**31**	8.968	4.766	0.575	0.693	0.181	2
Pop21	**30**	7.710	4.163	0.558	0.658	0.143	4
Pop22	**25**	8.645	4.556	0.548	0.669	0.171	2

Na: Number of Different Alleles; Ne: Number of Effective Alleles; Ho: Observed Heterozygosity; He: Expected Heterozygosity; F: Inbreeding coefficient.

The value of Na for the wild genotypes was significantly higher than that for the semi-wild genotypes (Table 3). The number of private alleles in the wild genotypes was far greater than that in the semi-wild genotypes. These differences could be associated with the huge disparities in sample size. The values of Ho and He for the wild genotypes were almost equal to the values for the semi-wild genotypes.

The Ho and He values in genetic cluster C3 were slightly larger than those in the other genetic clusters (Table 3) whereas genetic cluster C4 was the lowest Ho value; however, regardless of whether the individuals were considered to be wild or semi-wild, and regardless of whether they belonged to which genetic cluster, the Ho value was significantly lower than the He value. This result is in agreement with the high value of the fixation index, suggesting a deficit of heterozygotes with regard to the expectations of HWE.

A comparison of private alleles between the wild (152) and semi-wild (11) populations showed a significant difference between them (Table 3). When all populations were considered, P1 contained the most private alleles (7); no private alleles were found in P12 and P14.

### AMOVA

Our AMOVA revealed that a low percentage of variation was divided among natural populations, different origins, geographical distribution, and genetic clusters, respectively (Table 4). About 94% of the variation was attributed to differences within populations in all variance partitions. A hierarchical AMOVA of the four genetic clusters using STRUCTURE revealed that 1.87% of the variance was distributed among them, and it produced the largest F_ST_ value (0.06008). Seven genetic clusters revealed the highest percentage of variation (3.48%) among them, and it produced the second largest F_ST_ value (0.06002). With the populations grouped according to their geographical origin, a lower percentage of variation (1.98%) could be explained by geographic factors. When the populations were grouped according to their origin, a negative percentage of variation was detected among the groups.

**Table 4:** Analysis of molecular variance from microsatellite data using Arlequin version 3.5.

Source of variation	d.f.	Sum of squares	Variance components	Percentage of variation	Fixation Index
**Variance partition** a					
Among populations	21	956.633	**0.58429 Va**	5.59	F_ST_ = 0.05592
Within populations	1322	13039.582	**9.86353 Vb**	94.41	
Total	1343	13996.215	10.44782		
**Variance partition** b					
Among groups	1	24.409	−0.07047 Va	−0.68	F_ST_ = 0.05111
Among populations within groups	20	932.224	**0.60174 Vb**	5.79	F_SC_ = 0.05750
Within populations	1322	13039.582	**9.86353 Vc**	94.89	F_CT_ = −0.00678
Total	1343	13996.215	10.39480		
**Variance partition** c					
Among groups	5	390.503	**0.20726 Va**	1.98	F_ST_ = 0.05968
Among populations within groups	16	566.130	**0.41874 Vb**	3.99	F_SC_ = 0.04072
within populations	1322	13039.582	**9.86353 Vc**	94.03	F_CT_ = 0.01976
Total	1343	13996.215	10.48953		
**Variance partition** d					
Among groups	3	301.894	**0.19642 Va**	1.87	F_ST_ = 0.06008
Among populations within groups	18	654.739	**0.43402 Vb**	4.14	F_SC_ = 0.04215
within populations	1322	13039.582	**9.86353 Vc**	93.99	F_CT_ = 0.01872
Total	1343	13996.215	10.49397		
**Variance partition** e					
Among groups	6	511.653	**0.30467 Va**	3.48	F_ST_ = 0.06002
Among populations within groups	15	444.980	**0.32512 Vb**	2.43	F_SC_ = 0.03191
within populations	1322	13039.582	**9.86353 Vc**	94.10	F_CT_ = 0.02903
Total	1343	13996.215	10.49332		
**Variance partition** f					
Among groups	9	612.562	**0.30269 Va**	2.89	F_ST_ = 0.05844
Among populations within groups	12	344.071	**0.30951 Vb**	2.95	F_SC_ = 0.03042
within populations	1322	13039.582	**9.86353 Vc**	94.16	F_CT_ = 0.02889
Total	1343	13996.215	10.47573		
**Variance partition** g					
Among groups	13	746.937	**0.33358 Va**	3.19	F_ST_ = 0.05737
Among populations within groups	9	209.696	**0.26671 Vb**	2.55	F_SC_ = 0.02633
within populations	1322	13039.582	**9.86353 Vc**	94.26	F_CT_ = 0.03188
Total	1343	13996.215	10.46381		

The hierarchical analysis included 22 sampling populations in China including within populations, among populations within groups and among groups.

a The first analysis included all populations as one hierarchical group.

b The second analysis included two different origin groups.

c The third analysis included six geographical groups.

d The fourth analysis included four genetic clusters.

e The fifth analysis included seven genetic subclusters.

f The fifth analysis included ten genetic subclusters.

g The fifth analysis included fourteen genetic subclustersF_ST_ variance among coefficient of individual relative to the total variance.

F_SC_ variance among subpopulations within groups.

F_CT_ variance among groups relative to the total variance.

### Genetic and Geographic Relatedness

The pairwise genetic differentiation values (F_ST_ and R_ST_) calculated for the 22 populations showed genetic differentiation between each population (Table 5). All of the F_ST_ values were significantly different from 0 in all pairwise comparisons between the 22 populations (p<0.01). The lowest values of F_ST_ were observed between P1-P2, P1-P3, P1-P4, P1-P17, P2-P4, P4-P5, P4-P17, P5-P17, P11-P12 and P13-P14. Populations from different genetic clusters appeared to be more differentiated from each other, corresponding well to the classification of the genetic cluster. Of the R_ST_ values, 10 (8 of which were from paired populations coming from different clusters) were not significantly different from 0. The pairwise genetic differentiation values (F_ST_) between the four genetic clusters showed a higher genetic differentiation between three population pairs (C1-C2, C1-C4, and C3-C4) (Table S3).

**Table 5:** Pairwise F_ST_ values (below diagonal) and Pairwise R_ST_ values (above diagonal) between 22 populations.

	P1	P2	P3	P4	P5	P6	P7	P8	P9	P10	P11	P12	P13	P14	P15	P16	P17	P18	P19	P20	P21	P22
P1	0	0.033	0.015	0.038	0.024	0.095	0.038	0.021	0.045	0.085	0.049	0.042	0.048	0.043	0.090	0.106	0.058	0.097	0.023	0.080	0.009ns	0.007ns
P2	0.012	0	0.049	0.075	0.036	0.099	0.068	0.047	0.054	0.070	0.076	0.061	0.087	0.058	0.068	0.115	0.044	0.102	0.055	0.083	0.034	0.004ns
P3	0.018	0.021	0	0.057	0.035	0.124	0.081	0.057	0.054	0.096	0.044	0.055	0.071	0.061	0.074	0.101	0.082	0.129	0.011ns	0.091	0.015ns	0.017ns
P4	0.017	0.019	0.024	0	0.025	0.090	0.060	0.044	0.047	0.082	0.051	0.048	0.076	0.063	0.122	0.100	0.067	0.120	0.066	0.086	0.045	0.040
P5	0.026	0.024	0.034	0.015	0	0.075	0.052	0.021	0.045	0.053	0.056	0.037	0.081	0.037	0.091	0.061	0.043	0.072	0.039	0.063	0.025	0.003ns
P6	0.035	0.039	0.052	0.042	0.048	0	0.077	0.042	0.037	0.048	0.089	0.072	0.121	0.056	0.115	0.084	0.030	0.064	0.122	0.020	0.100	0.073
P7	0.051	0.057	0.060	0.052	0.053	0.031	0	0.027	0.055	0.072	0.054	0.021	0.052	0.025	0.109	0.097	0.039	0.082	0.075	0.079	0.051	0.052
P8	0.064	0.064	0.074	0.062	0.060	0.038	0.041	0	0.043	0.051	0.054	0.023	0.039	0.018	0.090	0.068	0.031	0.040	0.052	0.044	0.036	0.021
P9	0.044	0.047	0.058	0.043	0.037	0.051	0.040	0.059	0	0.059	0.033	0.043	0.089	0.045	0.092	0.063	0.020	0.120	0.059	0.038	0.055	0.027
P10	0.027	0.028	0.030	0.022	0.027	0.038	0.042	0.052	0.025	0	0.076	0.061	0.111	0.041	0.061	0.046	0.047	0.075	0.114	0.012ns	0.079	0.047
P11	0.029	0.039	0.045	0.031	0.042	0.049	0.051	0.069	0.039	0.026	0	0.054	0.073	0.041	0.085	0.076	0.046	0.136	0.047	0.072	0.034	0.036
P12	0.031	0.031	0.043	0.033	0.040	0.042	0.050	0.065	0.046	0.029	0.016	0	0.046	0.019	0.101	0.065	0.038	0.076	0.056	0.050	0.058	0.048
P13	0.028	0.037	0.040	0.029	0.045	0.046	0.056	0.072	0.044	0.023	0.038	0.037	0	0.034	0.083	0.112	0.091	0.082	0.077	0.108	0.052	0.067
P14	0.052	0.054	0.060	0.040	0.052	0.067	0.064	0.089	0.054	0.035	0.055	0.052	0.021	0	0.062	0.060	0.048	0.046	0.068	0.042	0.040	0.026
P15	0.056	0.059	0.058	0.048	0.064	0.064	0.082	0.084	0.069	0.050	0.058	0.054	0.047	0.057	0	0.083	0.076	0.096	0.091	0.085	0.060	0.059
P16	0.098	0.086	0.112	0.090	0.099	0.096	0.081	0.106	0.097	0.082	0.087	0.088	0.088	0.102	0.109	0	0.044	0.091	0.109	0.050	0.101	0.069
P17	0.017	0.024	0.027	0.014	0.019	0.037	0.044	0.062	0.031	0.023	0.027	0.031	0.023	0.033	0.047	0.089	0	0.082	0.066	0.040	0.061	0.034
P18	0.058	0.060	0.074	0.061	0.070	0.066	0.071	0.077	0.072	0.058	0.067	0.070	0.054	0.074	0.092	0.104	0.053	0	0.130	0.093	0.098	0.075
P19	0.025	0.024	0.041	0.035	0.040	0.029	0.048	0.063	0.046	0.030	0.033	0.032	0.034	0.050	0.062	0.080	0.026	0.038	0	0.100	0.010ns	0.036
P20	0.040	0.042	0.055	0.057	0.063	0.049	0.055	0.069	0.065	0.044	0.054	0.060	0.061	0.083	0.092	0.102	0.053	0.059	0.030	0	0.088	0.057
P21	0.066	0.068	0.080	0.079	0.083	0.077	0.080	0.092	0.085	0.069	0.081	0.088	0.074	0.103	0.110	0.102	0.078	0.052	0.046	0.050	0	0.009ns
P22	0.075	0.068	0.084	0.090	0.089	0.083	0.095	0.118	0.101	0.074	0.086	0.090	0.082	0.103	0.109	0.135	0.080	0.069	0.048	0.048	0.065	0

Significant values at the 1% nominal level are bolded.

**Figure 4:**
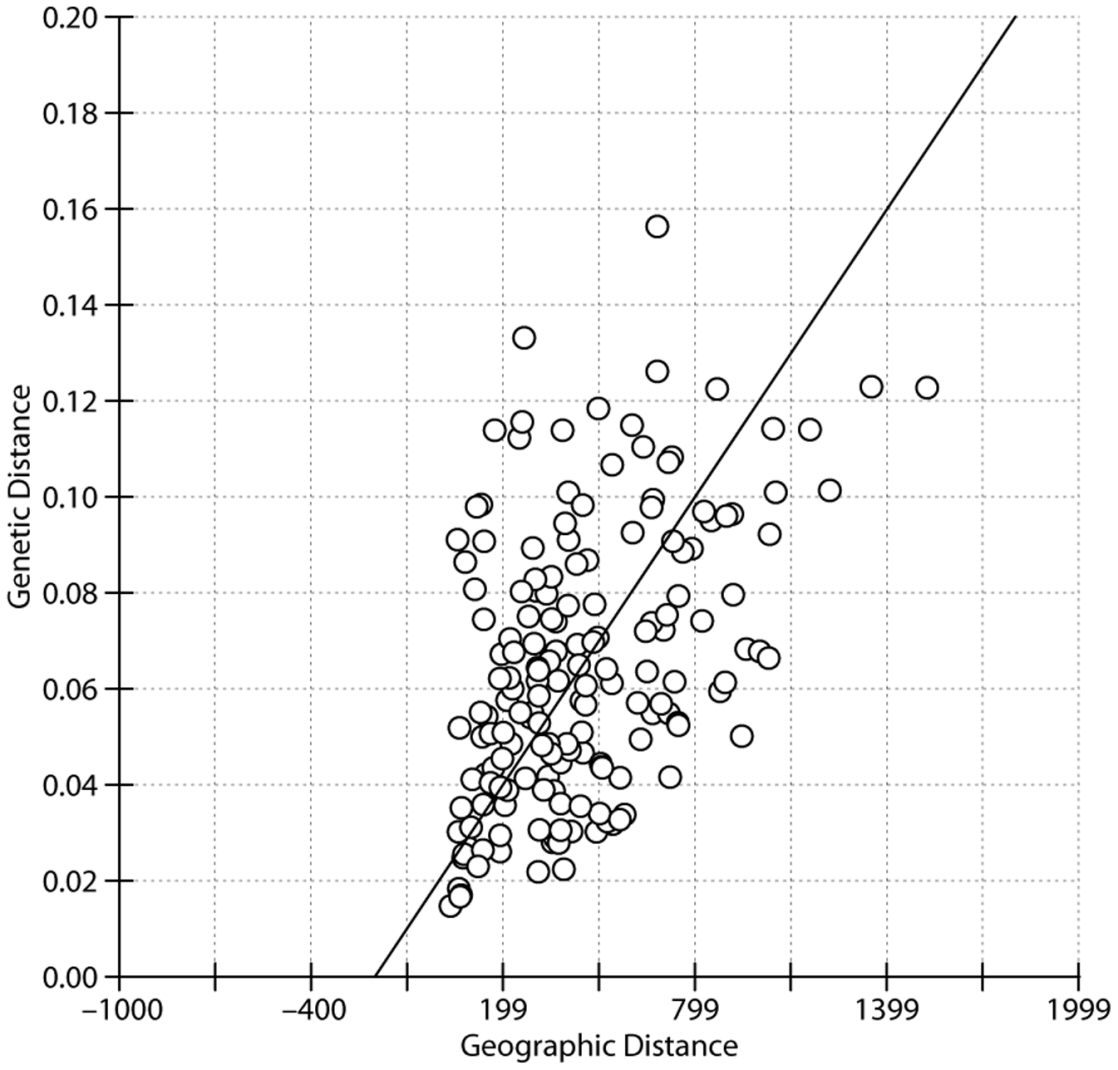
Correlation between genetic distance and geographic distance for Siberian apricot populations. Genetic distance is represented by pairwise F_ST_/(1–F_ST_) estimates among populations, which is regressed against the geographic distance. The RMA regression line overlays the scatterplot.

The highest F_ST_ values were observed for pairwise comparisons between P16 and other populations. The geographic distance matrix ranged from 35.8 to 1,526.5 km, based on the latitude and longitude values of all 19 wild populations. Rousset’s genetic distance values [F_ST_/(1–F_ST_)] [36] indicated that the most closely related Siberian apricot populations were P11 and P12, even though the geographical distance between them was not the closest. The greatest geographic distance (1,526.5 km) was between P15 and P16; however, this pairing did not have the largest Rousset’s distance (0.123). The largest Rousset’s distance (0.156) was between P16 and P22. The Mantel test (Figure 4) showed that genetic distance was not significantly correlated with geographic distance (r = 0.4651, p = 0.9940).

### The Identification of Genetic Barriers

**Figure 5:**
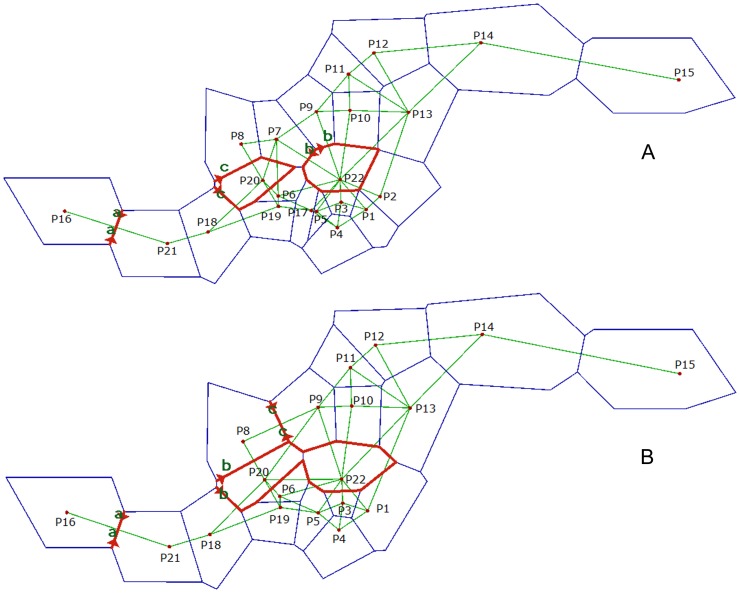
Genetic barriers predicted by BARRIER (version 2.2). The genetic barriers are shown in bold lines with arrows. A: genetic barrier predication using all populations; B: genetic barrier predication using all wild populations.

A genetic barrier prediction analysis using Monmonier’s maximum difference algorithm identified three putative barriers when all populations were included (Figure 5A). The first barrier separated the western peripheral population P16 from all other populations. The second predicted barrier separated population P22, which was located in the center of the distribution areas. The third predicted barrier separated population P20,. When only the 19 wild populations were included (Figure 5B), the first barriers separated P16, similar to the result obtained when all of the populations were included. The second predicted barrier separated P20 and P22 from the other populations. There was a gap between P20 and P22 that could be associated with each other. The second and the third predicted barriers together separated P8 from the other populations.

## Discussion

### Genetic Diversity of Siberian Apricot in China

Heterozygosity is an important measurement of gene diversity [40]. In our study, a relatively high level of genetic diversity was detected at microsatellite loci in Siberian apricot; the mean Ho and He values were 0.639 and 0.774, respectively. Similar values were reported for populations of Siberian apricot in the Yan Mountains (Ho = 0.668, He = 0.788) [15]. Fewer polymorphisms have been reported for apricot (*Prunus armenica* L.; Ho = 0.615, He = 0.621) [41]. The genetic diversity of Chinese wild almond (*Amygdalus nana* L.; Ho = 0.339, He = 0.219) is reportedly even lower [42]. Ferrer et al. [43] found that the number of loci and populations included in studies might affect estimates of genetic diversity. In our study, the number of loci and samples was larger than in the aforementioned studies. The geographic range of the species and species characteristics (e.g., long-lived, outcrossing, and wind-pollinated) also influenced the genetic diversity, and high heterozygosity could be favorable in long-lived plants growing in arid zones. Indeed, Siberian apricot is long-lived, wind-pollinated, self-incompatible, and distributed across a wide area with a harsh environment, which may be one cause of the high level of genetic diversity and high number of alleles of per loci detected in Siberian apricot populations. We have found many morphological variations in our field investigation, such as double petals apricot, green sepal apricot, big flower apricot, late flowering apricot, heart-shaped apricot, sweet benevolence apricot and so on, which have not been reported previously. Among the populations, P16 and P21 had the lowest level of genetic diversity (Table 3). P16 is located in the western edge of the distribution area whereas P21 is located 1,200 m above sea level at the southwestern edge of the Yan Mountains. The marginal distribution would reduce the opportunity to communicate with other populations and lead to a low level of genetic diversity.

The Ho value was lower than the He value at all 31 loci (Table 2), indicating a deficiency of heterozygotes at these loci. A heterozygote deficiency was also observed at the population level (Table 3). Similar findings related to heterozygote deficiency have been observed in other trees [44]–[46]. In *Cinnamomum insularimontanum* Hyata (*Lauraceae*) from southern Korea, a heterozygote deficiency was explained as a process of partial selfing rather than the presence of null alleles or a temporal Wahlund effect. A deficiency of heterozygotes in the tropical species *Sextonia rubra* (Mez) van der Werff was explained as an effect of biparental breeding due to limited pollen dispersal among relatives [45]. In flowering dogwood trees, a deficiency in heterozygotes was explained as the result of half-sibling mating occurring over a small geographical area [46]. The seed-setting rate by self-pollination in Siberian apricot is very low; such trees usually exhibit self-incompatibility. Thus, the deficiency of heterozygotes in Siberian apricot in our study may be the result of low levels of inbreeding. Further research on the mating system, pollen dispersal, and seeds in Siberian apricot populations is needed to infer the precise cause of the deficiency in heterozygotes.

### Genetic Structure of Siberian Apricot

An AMOVA revealed that genetic variation within populations accounted for about 94.4% of the total (Table 4). Outcrossing woody plants tend to be more genetically diverse and have less genetic differentiation among populations [32]. The percentage of genetic variation within populations of Siberian apricot in the Yan Mountains was shown to be up to 96% [15]. The negative percentage of variation detected among wild and semi-wild groups suggests that there is no significant difference between them. Furthermore, the values of Ne, He,Ho and F in the semi-wild population were similar to those in the wild population. This indicates that the sources of semi-wild populations might be selected randomly from the seeds of wild populations, and that recent cultivation practices have had little impact on the genetic diversity of Siberian apricot. The relatively low values of Na in the semi-wild group might be due to the small sample size.

The use of R- and F-statistics when estimating genetic differentiation assumes a stepwise-mutation model (SMM) and an infinite-allele model, respectively. R-statistics was developed to take into account the high homoplasy inherent in microsatellite markers [47]. However, several analyses of population structure have reached the conclusion that many microsatellite loci do not fit an SMM process [48]–[50]. Balloux et al. [51] showed that microsatellites could mutate following a fairly strict SMM model. De Andrés et al. [52] also chose F_ST_ instead of R_ST_ when calculating the genetic differentiation among grapevine populations. Compared with R_ST_, F_ST_ was more consistent with our other analysis. Though all the genetic differentiation between pairwise populations was significant, the lowest values of F_ST_ still could be observed between P1-P2, P1-P3, P1-P4, P1-P17, P2-P3, P2-P4, P4-P5, P4-P17, P5-P17, P11-P12 and P13-P14. An UPGMA dendrogram based on Nei’s unbiased genetic distance showed these population pairs had the shortest genetic distance (Figure S1). In addition, the clustering analysis showed these pairwise populations with low F_ST_ value clustered into a genetic cluster. All pairwise populations with the lowest genetic differentiation were from the same region (Figure 1), except P13-P14. We did not find variation in the Siberian apricot trees around P13 and P14, indicating that they are isolated populations. The distance from P13 to P14 is about 300 km, which is far enough that the two populations have little chance to exchange genes. The F_ST_ value between P13 and P14 (Table 5) is not significantly different, suggesting a low degree of genetic differentiation between them. It may be that a long time ago human activity severed the continuity of their distribution, but that the later development of a similar environment at the two sampling locations (the eastern edge of the Greater Khingan Mountains and western edge of the Northeast Plain) guided the evolution of the two populations in the same direction. Isolated populations cannot communicate with outside populations, which may increase the chance of inbreeding. The relatively high positive value of F (Table 3) supports this possibility.

STRUCTURE has been successfully used in a large variety of population genetic studies, including in studies of genetic structure, the distinguishing of breeds, and the detection of hybrids between cultivated and wild assortments [53]–[55]. In general, two models are used to identify the true optimum number of subsets (K) in STRUCTURE. The first model, described by Pritchard et al. [28], is based on the probability Pr(X|K) (called Ln P[D] in STRUCTURE), and the K value that provides the maximum Ln P(D) value is selected as the optimum number of subsets [56]. Evanno et al. [31] found that in many cases the estimated Ln P(D) does not help visualize the correct number of clusters (K). They recommended using an ad hoc statistic, ΔK, based on the rate of change in the log probability of data between successive K values evaluated by STRUCTURE to more accurately detect the real number of clusters [57], [58]. However, Vigouroux et al. [59] pointed out that the ΔK method of Evanno et al. [31] always favored K = 2 in the main structure analysis. When large datasets are analyzed, a convergence problem for the Gibbs sampler algorithm used in STRUCTURE may occur [60], [61]. Recently, Jacobs et al. [62] grouped populations by maximizing the allocation of genetic diversity among subgroups (i.e., maximizing the F_ST_ values). This provided a new means of identifying the true optimum number of subsets. The result of AMOVA showed that the maximum F_ST_ value (0.06008) when all populations were grouped into four genetic clusters (Table 4).

In this study, STRUCTURE identified four main genetic groups (clusters) (Table S2). All genetic clusters showed high average ancestry values, as compared to their own clusters. The populations from G3 were almost all clustered into genetic cluster C1, while the populations from G1 were clustered into genetic cluster C4 except P6. However, based on the ancestry values of all of the individuals (Table S2), we found that a high number of individuals from P9 (2 individuals), P10 (2 individuals), P11 (2 individuals), P12 (6 individuals), P18 (1 individual), and P19 (5 individuals), belonged to the other genetic clusters, which were clustered in genetic cluster C1. Similar results were also found in the other genetic clusters. Siberian apricot reproduces mainly by seeds from ripe and dehiscent fruits. A natural gene flow over such distance could not be possible, and one putative explanation could be dispersal by some kinds of rodent and human actions. The Korean field mouse (*Apodemus peninsulae*), whitebellied rat (*Niviventer confucianus*), striped field mouse (*Apodemus agrarius*), and other rodents feed and store Siberian apricot seeds, which makes long-distance gene flow possible and improves the level of genetic diversity.

According to the results of our structure analysis, most of the populations that were geographically close were generally clustered into the same cluster. An analysis based on the Mantel test (Figure 4) showed that the genetic distance was not significantly correlated with the geographic distance (r = 0.4651, p = 0.9940), suggesting that geographic distance is not the principal factor influencing genetic differentiation in Siberian apricot. The distance between P5 and P17 was <17 km; however, the populations were not clustered into the same cluster when K>4. Furthermore, significant genetic differentiation was detected between them (Table 5), suggesting that the seeds from the semi-wild population in P17 was not from the local.

P16 was separated by the first predicted barriers, regardless of whether the three semi-wild populations were included or not (Figure 5). However, the second and third predicted barriers produced different results. If semi-wild populations were excluded, P22 had an exchange with P20 (belonging to genetic cluster C4) while P8 did not exchange with other populations. It is possible that the seed resources of P7 were from P8, because they were from the same genetic cluster when K = 14, and the seed resources of P17 were from genetic cluster C1 and genetic cluster C3. Most of the populations with low-level genetic diversity (Table 3) were separated from other populations, indicating that the barrier was an important factor influencing genetic diversity. Further investigation into how these genetic barriers are related to geographic or other factors is needed.

## Conclusions

Our studies show a relatively high level of genetic diversity among Siberian apricot populations in China. However, a significant deficiency in heterozygotes was detected at the locus and population levels, which may be the result of low-level inbreeding. Our structure analysis clustered all of the populations into four genetic clusters. There was no significant difference between the wild and semi-wild groups, indicating that recent cultivation practices have had little impact on the genetic diversity of Siberian apricot. Our study represents the most comprehensive investigation of the genetic diversity and population structure of Siberian apricot in China and will provide valuable information for the collection of genetic resources for the breeding of Siberian apricot and related species.

## Supporting Information

Figure S1 **UPGMA dendrogram of Siberian apricot populations based on Nei’s unbiased genetic distance.**
(TIF)

Table S1 **Primer information for 31 microsatellite loci used to analyze 672 Siberian apricot samples.**
(DOC)

Table S2 **Mean ancestry values for the four genetic groups inferred by STRUCTURE.**
(XLS)

Table S3 **Pairwise estimates of FST values based on data from 31 SSR loci among the model-based clusters inferred by Structure.** Significant values at the 1% nominal level are bolded.
(DOC)

## References

[1] Wang LB (2011) Resource Investigation and Distribution Pattern of Three Armeniaca Species. Forest Resources Management 5: 65–70.

[2] Gumus M, Kasifoglu S (2010) Performance and emission evaluation of a compression ignition engine using a biodiesel (apricot seed kernel oil methyl ester) and its blends with diesel fuel. Biomass and bioenergy 34: 134–139.

[3] Wang LB (2012) Evaluation of Siberian Apricot (*Prunus sibirica* L.) Germplasm Variability for Biodiesel Properties. Journal of the American Oil Chemists’ Society 89: 1743–1747.

[4] Li XF, Liu MY, Guo XY (2005) Introduction on sustainable management and development of wild Siberian apricot. Inner Mongolia Forestry Investigation and Design 28: 21–22.

[5] Wang ZM, Feng LJ, Feng CF, Liu LQ (2001) A diseases and insect survey report of Siberian apricot in Cayouzhongqi. Inner Mongolia Forestry Investigation and Design 24: 35–36.

[6] Ali M, Rajewski J, Baenziger P, Gill K, Eskridge K, et al (2008) Assessment of genetic diversity and relationship among a collection of US sweet sorghum germplasm by SSR markers. Molecular Breeding 21: 497–509.

[7] Bovine HapMap C, Gibbs RA, Taylor JF, Van Tassell CP, Barendse W, et al (2009) Genome-wide survey of SNP variation uncovers the genetic structure of cattle breeds. Science 324: 528–532. 19390050 10.1126/science.1167936 PMC2735092

[8] Hagen S, Khadari B, Lambert P, Audergon JM (2002) Genetic diversity in apricot revealed by AFLP markers: species and cultivar comparisons. Theor Appl Genet 105: 298–305. 12582532 10.1007/s00122-002-0910-8

[9] Sánchez-Pérez R, Martínez-Gómez P, Dicenta F, Egea J, Ruiz D (2006) Level and transmission of genetic heterozygosity in apricot (*Prunus armeniaca* L.) explored using simple sequence repeat markers. Genetic Resources and Crop Evolution 53: 763–770.

[10] Vicente Md, Truco M, Egea J, Burgos L, Arús P (1998) RFLP variability in apricot (*Prunus armeniaca* L.). Plant Breeding 117: 153–158.

[11] Kijas J, Fowler J, Thomas M (1995) An evaluation of sequence tagged microsatellite site markers for genetic analysis within *Citrus* and related species. Genome 38: 349–355. 7774802 10.1139/g95-045

[12] Du Q, Wang B, Wei Z, Zhang D, Li B (2012) Genetic diversity and population structure of Chinese White poplar (*Populus tomentosa*) revealed by SSR markers. J Hered 103: 853–862. 23008443 10.1093/jhered/ess061

[13] Hormaza JI (2002) Molecular characterization and similarity relationships among apricot (*Prunus armeniaca* L.) genotypes using simple sequence repeats. Theor Appl Genet 104: 321–328. 12582704 10.1007/s001220100684

[14] Zhebentyayeva TN, Reighard GL, Gorina VM, Abbott AG (2003) Simple sequence repeat (SSR) analysis for assessment of genetic variability in apricot germplasm. Theor Appl Genet 106: 435–444. 12589543 10.1007/s00122-002-1069-z

[15] Liu HB, Wang Z, Liu J, Ma LY, Wang SQ, et al (2012) Genetic Diversity and Genetic Structure of Siberian Apricot Populations in the Yan Mountains. Scientia Silvae Sinicae 48: 68–74.

[16] Liu HB, Liu J, Wang Z, Ma LY, Wang SQ, et al (2013) Development and Characterization of Microsatellite Markers in *Prunus sibirica* (*Rosaceae*). Applications in Plant Sciences 1: 1200074. 10.3732/apps.1200074 PMC4105279 25202522

[17] Doyle JJ (1987) A rapid DNA isolation procedure for small quantities of fresh leaf tissue. Phytochem bull 19: 11–15.

[18] Wang Z, Liu HB, Liu J, Li YY, Wu RL, et al (2014) Mining new microsatellite markers for Siberian apricot (Prunus sibirica L.) from SSR-enriched genomic library. Scientia Horticulturae 166: 65–69.

[19] Lopes M, Sefc K, Laimer M, Da Câmara Machado A (2002) Identification of microsatellite loci in apricot. Molecular Ecology Notes 2: 24–26. 10.1046/j.1365-294x.2000.00954.x 10964237

[20] Aranzana M, Garcia-Mas J, Carbo J, Arús P (2002) Development and variability analysis of microsatellite markers in peach. Plant Breeding 121: 87–92.

[21] Dirlewanger E, Cosson P, Tavaud M, Aranzana J, Poizat C, et al (2002) Development of microsatellite markers in peach [Prunus persica (L.) Batsch] and their use in genetic diversity analysis in peach and sweet cherry (Prunus avium L.). Theor Appl Genet 105: 127–138. 12582570 10.1007/s00122-002-0867-7

[22] Testolin R, Marrazzo T, Cipriani G, Quarta R, Verde I, et al (2000) Microsatellite DNA in peach (*Prunus persica* L. Batsch) and its use in fingerprinting and testing the genetic origin of cultivars. Genome 43: 512–520. 10902716

[23] Chen Q, Zhang L, Yuan Z, Yan Z, Zheng Y, et al (2008) Empirical verification of heterogeneous DNA fragments generated from wheat genome-specific SSR primers. Canadian Journal of Plant Science 88: 1065–1071.

[24] Amos W, Hoffman J, Frodsham A, Zhang L, Best S, et al (2007) Automated binning of microsatellite alleles: problems and solutions. Molecular Ecology Notes 7: 10–14.

[25] Park SDE (2001) Trypanotolerance in West African cattle and the population genetic effects of selection. Ph D thesis, University of Dublin.

[26] Peakall R, Smouse PE (2006) Genalex 6: genetic analysis in Excel. Population genetic software for teaching and research. Molecular Ecology Notes 6: 288–295. 10.1093/bioinformatics/bts460 PMC3463245 22820204

[27] Nei M (1973) Analysis of gene diversity in subdivided populations. Proc Natl Acad Sci U S A 70: 3321–3323. 4519626 10.1073/pnas.70.12.3321 PMC427228

[28] Pritchard JK, Stephens M, Donnelly P (2000) Inference of population structure using multilocus genotype data. Genetics 155: 945–959. 10835412 10.1093/genetics/155.2.945 PMC1461096

[29] Pritchard JK, Donnelly P (2001) Case-control studies of association in structured or admixed populations. Theor Popul Biol 60: 227–237. 11855957 10.1006/tpbi.2001.1543

[30] Hubisz MJ, Falush D, Stephens M, Pritchard JK (2009) Inferring weak population structure with the assistance of sample group information. Mol Ecol Resour 9: 1322–1332. 21564903 10.1111/j.1755-0998.2009.02591.x PMC3518025

[31] Evanno G, Regnaut S, Goudet J (2005) Detecting the number of clusters of individuals using the software Structure: a simulation study. Mol Ecol 14: 2611–2620. 15969739 10.1111/j.1365-294X.2005.02553.x

[32] Hamrick J, Godt M (1996) Effects of life history traits on genetic diversity in plant species. Philosophical Transactions of the Royal Society of London Series B: Biological Sciences 351: 1291–1298.

[33] Excoffier L, Lischer HE (2010) Arlequin suite ver 3.5: a new series of programs to perform population genetics analyses under Linux and Windows. Mol Ecol Resour 10: 564–567. 21565059 10.1111/j.1755-0998.2010.02847.x

[34] Guo SW, Thompson EA (1992) Performing the exact test of Hardy-Weinberg proportion for multiple alleles. Biometrics 48: 361–372. 1637966

[35] Goudet J (2001) FSTAT, a program to estimate and test gene diversities and fixation indices (version 2.9. 3).

[36] Rousset F (1997) Genetic differentiation and estimation of gene flow from F-statistics under isolation by distance. Genetics 145: 1219–1228. 9093870 10.1093/genetics/145.4.1219 PMC1207888

[37] Jensen JL, Bohonak AJ, Kelley ST (2005) Isolation by distance, web service. BMC Genet 6: 13. 15760479 10.1186/1471-2156-6-13 PMC1079815

[38] Mantel N (1967) Ranking procedures for arbitrarily restricted observation. Biometrics 23: 65–78. 6050473

[39] Manni F, Guerard E, Heyer E (2004) Geographic patterns of (genetic, morphologic, linguistic) variation: how barriers can be detected by using Monmonier’s algorithm. Hum Biol 76: 173–190. 15359530 10.1353/hub.2004.0034

[40] Slatkin M, Barton NH (1989) A comparison of three indirect methods for estimating average levels of gene flow. Evolution: 1349–1368. 10.1111/j.1558-5646.1989.tb02587.x 28564250

[41] Donoso J, Aros D, Meneses C, Narváez C, Infante R, et al (2008) Genetic relationships in apricot (*Prunus armeniaca* L.) using SSR markers and their implications for breeding. Journal of Food Agriculture & Environment 6: 378–382.

[42] Tahan O, Geng Y, Zeng L, Dong S, Chen F, et al (2009) Assessment of genetic diversity and population structure of Chinese wild almond, *Amygdalus nana*, using EST-and genomic SSRs. Biochemical Systematics and Ecology 37: 146–153.

[43] Ferrer MM, Eguiarte LE, Montana C (2004) Genetic structure and outcrossing rates in *Flourensia cernua* (*Asteraceae*) growing at different densities in the South-western Chihuahuan Desert. Ann Bot 94: 419–426. 15277246 10.1093/aob/mch159 PMC4242184

[44] Chung MY, Nason JD, Epperson BK, Chung MG (2003) Temporal aspects of the fine-scale genetic structure in a population of *Cinnamomum insularimontanum* (*Lauraceae*). Heredity (Edinb) 90: 98–106. 12522432 10.1038/sj.hdy.6800187

[45] Veron V, Caron H, Degen B (2005) Gene flow and mating system of the tropical tree *Sextonia rubra* . Silvae genetica 54: 275–280.

[46] Hadziabdic D, Wang X, Wadl PA, Rinehart TA, Ownley BH, et al (2012) Genetic diversity of flowering dogwood in the Great Smoky Mountains National Park. Tree Genetics & Genomes 8: 855–871.

[47] Slatkin M (1995) A measure of population subdivision based on microsatellite allele frequencies. Genetics 139: 457–462. 7705646 10.1093/genetics/139.1.457 PMC1206343

[48] Estoup A, Garnery L, Solignac M, Cornuet JM (1995) Microsatellite variation in honey bee (*Apis mellifera* L.) populations: hierarchical genetic structure and test of the infinite allele and stepwise mutation models. Genetics 140: 679–695. 7498746 10.1093/genetics/140.2.679 PMC1206644

[49] Goodman SJ (1998) Patterns of extensive genetic differentiation and variation among European harbor seals (*Phoca vitulina vitulina*) revealed using microsatellite DNA polymorphisms. Mol Biol Evol 15: 104–118. 9491609 10.1093/oxfordjournals.molbev.a025907

[50] Ross KG, Krieger MJ, Shoemaker DD, Vargo EL, Keller L (1997) Hierarchical analysis of genetic structure in native fire ant populations: results from three classes of molecular markers. Genetics 147: 643–655. 9335601 10.1093/genetics/147.2.643 PMC1208186

[51] Balloux F, Brunner H, Lugon-Moulin N, Hausser J, Goudet J (2000) Microsatellites can be misleading: an empirical and simulation study. Evolution 54: 1414–1422. 11005307 10.1111/j.0014-3820.2000.tb00573.x

[52] De Andres MT, Benito A, Perez-Rivera G, Ocete R, Lopez MA, et al (2012) Genetic diversity of wild grapevine populations in Spain and their genetic relationships with cultivated grapevines. Mol Ecol 21: 800–816. 22151598 10.1111/j.1365-294X.2011.05395.x

[53] Pineiro R, Fuertes Aguilar J, Munt DD, Nieto Feliner G (2007) Ecology matters: Atlantic-Mediterranean disjunction in the sand-dune shrub Armeria pungens (*Plumbaginaceae*). Mol Ecol 16: 2155–2171. 17498238 10.1111/j.1365-294X.2007.03280.x

[54] Coart E, Vekemans X, Smulders MJ, Wagner I, Van Huylenbroeck J, et al (2003) Genetic variation in the endangered wild apple (*Malus sylvestris* (L.) Mill.) in Belgium as revealed by amplified fragment length polymorphism and microsatellite markers. Mol Ecol 12: 845–857. 12753206 10.1046/j.1365-294x.2003.01778.x

[55] Koopman WJ, Li Y, Coart E, van de Weg WE, Vosman B, et al (2007) Linked vs. unlinked markers: multilocus microsatellite haplotype-sharing as a tool to estimate gene flow and introgression. Molecular ecology 16: 243–256. 17217342 10.1111/j.1365-294X.2006.03137.x

[56] López-Gartner G, Cortina H, McCouch SR, Moncada MDP (2009) Analysis of genetic structure in a sample of coffee (*Coffea arabica* L.) using fluorescent SSR markers. Tree Genetics & Genomes 5: 435–446.

[57] Krutovsky KV, Clair JBS, Saich R, Hipkins VD, Neale DB (2009) Estimation of population structure in coastal Douglas-fir [*Pseudotsuga menziesii* (Mirb.) Franco var. *menziesii*] using allozyme and microsatellite markers. Tree Genetics & Genomes 5: 641–658.

[58] D’Hoop BB, Paulo MJ, Kowitwanich K, Sengers M, Visser RG, et al (2010) Population structure and linkage disequilibrium unravelled in tetraploid potato. Theor Appl Genet 121: 1151–1170. 20563789 10.1007/s00122-010-1379-5 PMC2938457

[59] Vigouroux Y, Glaubitz JC, Matsuoka Y, Goodman MM, Sanchez GJ, et al (2008) Population structure and genetic diversity of New World maize races assessed by DNA microsatellites. Am J Bot 95: 1240–1253. 21632329 10.3732/ajb.0800097

[60] Rosenberg NA, Pritchard JK, Weber JL, Cann HM, Kidd KK, et al (2002) Genetic structure of human populations. Science 298: 2381–2385. 12493913 10.1126/science.1078311

[61] Corander J, Marttinen P, Siren J, Tang J (2008) Enhanced Bayesian modelling in BAPS software for learning genetic structures of populations. BMC Bioinformatics 9: 539. 19087322 10.1186/1471-2105-9-539 PMC2629778

[62] Jacobs MM, Smulders MJ, van den Berg RG, Vosman B (2011) What’s in a name; Genetic structure in *Solanum* section *Petota* studied using population-genetic tools. BMC evolutionary biology 11: 42. 21310063 10.1186/1471-2148-11-42 PMC3045909

