# Supplementary figures and images for: High-Level Genetic Diversity and Complex Population Structure of Siberian Apricot (Prunus sibirica L.) in China as Revealed by Nuclear SSR Markers
Source: PLoS One. 2014 Feb 7;9(2):e87381. doi: 10.1371/journal.pone.0087381 (PMC3917850; doi:10.1371/journal.pone.0087381)

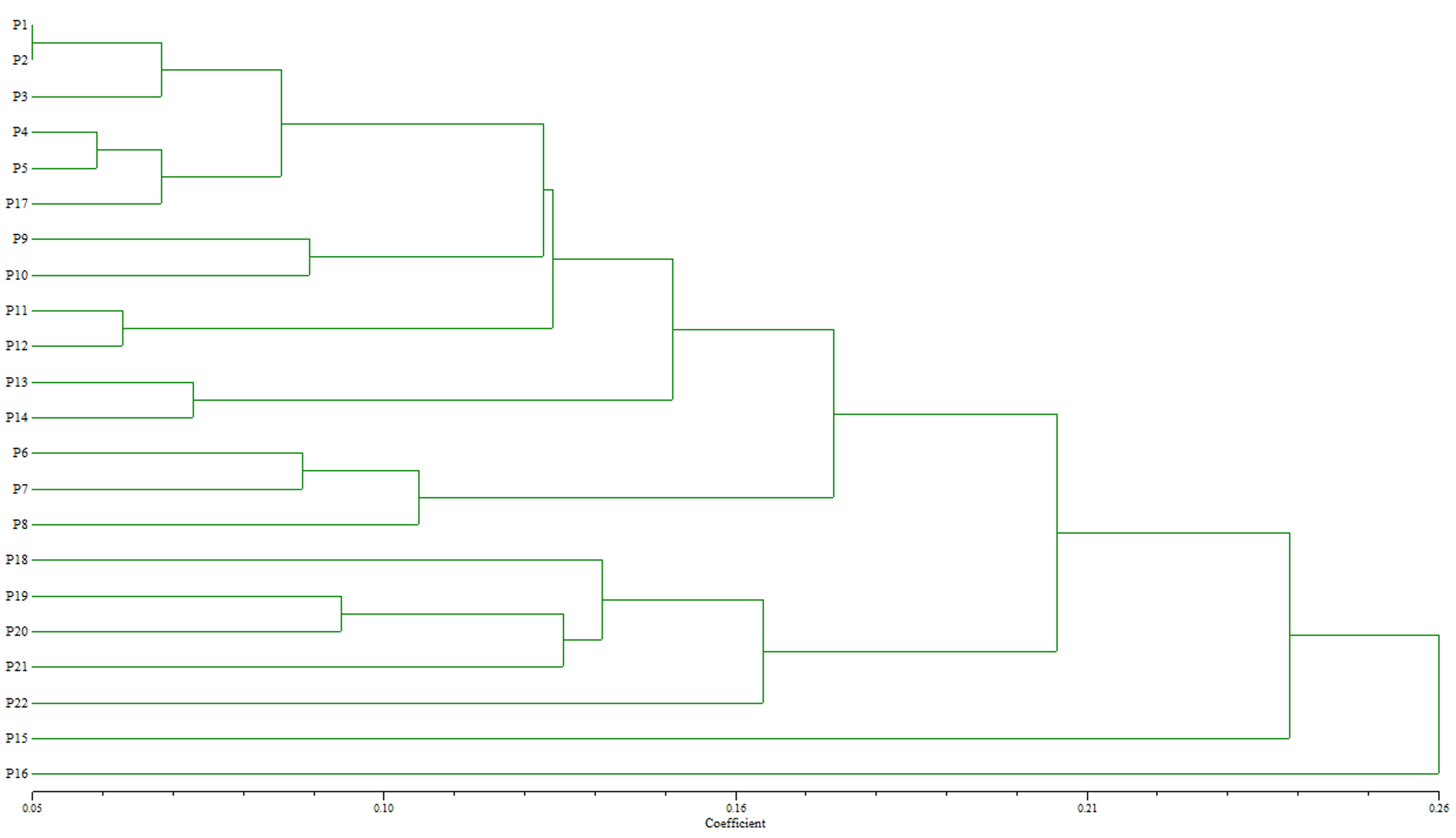

Supplement: Figure S1 — UPGMA dendrogram of Siberian apricot populations based on Nei’s unbiased genetic distance. (TIF) [file pone.0087381.s001.tif]
