# Supplementary material for: High-Level Genetic Diversity and Complex Population Structure of Siberian Apricot (Prunus sibirica L.) in China as Revealed by Nuclear SSR Markers
Source: PLoS One. 2014 Feb 7;9(2):e87381. doi: 10.1371/journal.pone.0087381 (PMC3917850; doi:10.1371/journal.pone.0087381)
Supplement: Table S1 — Primer information for 31 microsatellite loci used to analyze 672 Siberian apricot samples. (DOC) [file pone.0087381.s002.doc]

**Table S1.** Primer information for 31 microsatellite loci used to analyze 672 Siberian apricot samples.

| **Locus** | **Primer sequence (5'-3')** | | **Repeat motif** | **Size(bp)** | **T_a_(℃)** | **Source** | **Chromosome** |
| --- | --- | --- | --- | --- | --- | --- | --- |
| PSL1 | F: | GTGTGTTGGAGCCTTCCAGT | (AG)7 | 173 | 55 | Siberian apricot | - |
|  | R: | TCCTTCGTTTCCTTTTGTGC |  |  |  |
| PSL3 | F: | TCTCTTCTTTCGCTCGCTCT | (TCTTT)3 | 200 | 55 | Siberian apricot | - |
|  | R: | GGGTGCCCAGATCAGAAATA |  |  |  |
| PSL6 | F: | GTTCAAATGGTCCTCGCATT | (GA)13 | 180 | 55 | Siberian apricot | - |
|  | R: | TCTTGTGTTATCCGTCCGTTT |  |  |  |
| PSL7 | F: | TTTGGAGGTGGAAGAGGATG | (AG)12 | 151 | 55 | Siberian apricot | - |
|  | R: | CTTTGGCCCTCACAACAAGT |  |  |  |
| PSL8 | F: | AAGCAGGCTCTAACCAAGCA | (AG)9 | 218 | 55 | Siberian apricot | - |
|  | R: | TCCTTTAGTGGCACCCAGAC |  |  |  |
| PSL10 | F: | CCCATGGTATAGAGGATCCAGA | (TC)13 | 295 | 55 | Siberian apricot | - |
|  | R: | TGGCGAGAAGAAACCCTAGA |  |  |  |
| PSL11 | F: | CCATAACCAAAATGGAGAGCTT | (GA)6 | 221 | 55 | Siberian apricot | - |
|  | R: | CCTTGCATGACTTTCCCTTC |  |  |  |
| PSL12 | F: | CACCCCCAACATACCACTTC | (TC)7 | 143 | 55 | Siberian apricot | - |
|  | R: | GTGCTGCAAAAGCAAAAACA |  |  |  |
| PSL13 | F: | TTCGTCAGTTGGCTTCTCCT | (CT)12 | 293 | 55 | Siberian apricot | - |
|  | R: | CAGTCAAGAGCAGCTGCAAG |  |  |  |
| PSL14 | F: | AACCCACATTCCCATGCTTA | (GT)7 | 207 | 55 | Siberian apricot | - |
|  | R: | GCATCCGAGTTTGAGAGACG |  |  |  |
| PSL16 | F: | TCGATCAATCAAGGGCAACT | (GA)10 | 167 | 55 | Siberian apricot | - |
|  | R: | AACGTATGCCATCGTACCG |  |  |  |
| PSL18 | F: | GCCTTAGCTTTCTCTTCTTGGA | (GA)21 | 150 | 55 | Siberian apricot | - |
|  | R: | CCCTGTATAAACATCCCCCTTA |  |  |  |
| A1-10 | F: | AACCCCTCCTGTATCCCTTG | (AG)17 | 219 | 60 | Siberian apricot | - |
|  | R: | CGAATTTGAAGGTTGGGTTC |  |  |  |
| A3-9 | F: | CATGGAATGCTTTGTGATGG | (CT)8 | 235 | 60 | Siberian apricot | - |
|  | R: | CGGTGGGAAACTAAGGTGAA |  |  |  |
| A3-66 | F: | TCACAAACCCTCTGCTTTCA | (CT)15 | 230 | 60 | Siberian apricot | - |
|  | R: | TTCGGGGAACATAAACAAGC |  |  |  |
| H1-7 | F: | GGGATGATGCCACAGATTTT | (GA)11 | 292 | 60 | Siberian apricot | - |
|  | R: | GCTCACCACGTAGACGTTTC |  |  |  |
| H1-11 | F: | GTCCACGTGATCAAGCTCAG | (AG)17 | 130 | 60 | Siberian apricot | - |
|  | R: | TGTAAGCAACGAAACGGTCA |  |  |  |
| H1-77 | F: | AAGTACAACAGCGCCACTGC | (AG)8 | 150 | 60 | Siberian apricot | - |
|  | R: | CATCAACTTCGGCTCAGGAT |  |  |  |
| H1-87 | F: | CCTTGCATGACTTTCCCTTC | (TC)7 | 182 | 60 | Siberian apricot | - |
|  | R: | TGAACCATTGAGCCACAGAG |  |  |  |
| H2-11 | F: | TCAGAGGGATTGCTTTCCAC | (TGC)6 | 146 | 60 | Siberian apricot | - |
|  | R: | TCCTCCCAGCAGTCTCTTTC |  |  |  |
| H2-27 | F: | GTGTGGGAGACTCCTGCATT | (AG)17 | 158 | 60 | Siberian apricot | - |
|  | R: | ACTGACCAGAAGCCTGCATT |  |  |  |
| H2-77 | F: | CATGCACTTCACCCTCAATG | (GA)23 | 203 | 60 | Siberian apricot | - |
|  | R: | TAGAGCTGCCCTCCAACCTA |  |  |  |
| H2-79 | F: | TCTGCTTTTCGCTTCCAGTT | (AG)11 | 218 | 60 | Siberian apricot | - |
|  | R: | CCTCGCCATTAAAGAGCAAC |  |  |  |
| BPPCT 002 | F: | TCGACAGCTTGATCTTGACC | (AG)25 | 229 | 57~60 | Peach | G2 |
|  | R: | CAATGCCTA CGGAGATAAAAGAC |  |  |  |  |
| BPPCT 030 | F: | AATTGTACTTGCCAATGCTAT GA | (AG)25 | 175 | 57~60 | Peach | G2 |
|  | R: | CTGCCTTCTGCTCACACC |  |  |  |  |
| CPPCT6 | F: | TGTAAAACGACGGCCAGTAATTAACTCCAACAGCTCCA | (CT)16 | 190 | 59 | Peach | G8 |
|  | R: | ATGGTTGCTTAATTCAATGG |  |  |  |  |
| UDP96-001 | F: | AGTTTGATTTTCTGATGCATCC | (CA)17 | 120 | 57~63 | Peach | G6 |
|  | R: | TGCCATAAGGACCGGTATGT |  |  |  |  |
| UDP96-005 | F: | GTAACGCTCGCTACCACAAA | (AC)16TG(CT)2CA(CT)11 | 155 | 57~63 | Peach | G1 |
|  | R: | CACCCAGCTCATACACCTCA |  |  |  |
| UDP96-010 | F: | CCCATGTGTGTCCACATCTC | (GT)21(GAGT)4(GA)18 | 131 | 57~63 | Peach | G6 |
|  | R: | TTGATGATTCCATGCGTCTC |  |  |  |
| UDP98-412 | F: | AGGGAAAGTTTCTGCTGCAC | (AG)28 | 129 | 57~63 | Peach | G6 |
|  | R: | GCTGAAGACGACGATGATGA |  |  |  |  |
| ssrPaCITA15 | F: | GAGATTTGCAATGTCGGAATAAGAC | (TC)15 | 254 | 66 | Apricot | - |
|  | R: | CAGACAGCTGCTGGTTATAGGCTCG |  |  |  |  |

Note: “-“indicates not available
