# Supplementary material for: High-Level Genetic Diversity and Complex Population Structure of Siberian Apricot (Prunus sibirica L.) in China as Revealed by Nuclear SSR Markers
Source: PLoS One. 2014 Feb 7;9(2):e87381. doi: 10.1371/journal.pone.0087381 (PMC3917850; doi:10.1371/journal.pone.0087381)
Supplement: Table S3 — Pairwise estimates of FST values based on data from 31 SSR loci among the model-based clusters inferred by Structure. Significant values at the 1% nominal level are bolded. (DOC) [file pone.0087381.s004.doc]

|  | Genetic cluster 1 | Genetic cluster 2 | Genetic cluster 3 | Genetic cluster 4 |
| --- | --- | --- | --- | --- |
| Genetic cluster 1 | 0 |  |  |  |
| Genetic cluster 2 | 0.0315 | 0 |  |  |
| Genetic cluster 3 | 0.0127 | 0.0280 | 0 |  |
| Genetic cluster 4 | 0.0302 | 0.0338 | 0.0287 | 0 |

**Table S3.** Pairwise estimates of F_ST_ values based on data from 31 SSR loci among the model-based clusters inferred by Structure. Significant values at the 1% nominal level are bolded.
